# Matrix metalloproteinase-25 has a functional role in mouse secondary palate development and is a downstream target of TGF-β3

**DOI:** 10.1186/1471-213X-10-93

**Published:** 2010-09-01

**Authors:** Graham D Brown, Adil J Nazarali

**Affiliations:** 1 Laboratory of Molecular Biology, College of Pharmacy and Nutrition, University of Saskatchewan, Saskatoon, Saskatchewan, S7N 5C9, Canada

## Abstract

**Background:**

Development of the secondary palate (SP) is a complex event and abnormalities during SP development can lead to cleft palate, one of the most common birth disorders. Matrix metalloproteinases (MMPs) are required for proper SP development, although a functional role for any one MMP in SP development remains unknown. MMP-25 may have a functional role in SP formation as genetic scans of the DNA of human cleft palate patients indicate a common mutation at a region upstream of the MMP-25 gene. We report on the gene expression profile of MMP-25 in the developing mouse SP and identify its functional role in mouse SP development.

**Results:**

MMP-25 mRNA and protein are found at all SP developmental stages in mice, with the highest expression at embryonic day (E) 13.5. Immunohistochemistry and *in situ *hybridization localize MMP-25 protein and mRNA, respectively, to the apical palate shelf epithelial cells and apical mesenchyme. MMP-25 knockdown with siRNA in palatal cultures results in a significant decrease in palate shelf fusion and persistence of the medial edge epithelium. MMP-25 mRNA and protein levels significantly decrease when cultured palate shelves are incubated in growth medium with 5 μg/mL of a TGF-β3-neutralizing antibody.

**Conclusions:**

Our findings indicate: (i) MMP-25 gene expression is highest at E12.5 and E13.5, which corresponds with increasing palate shelf growth downward alongside the tongue; (ii) MMP-25 protein and mRNA expression predominantly localize in the apical epithelium of the palate shelves, but are also found in apical areas of the mesenchyme; (iii) knockdown of MMP-25 mRNA expression impairs palate shelf fusion and results in significant medial edge epithelium remaining in contacted areas; and (iv) bio-neutralization of TGF-β3 significantly decreases MMP-25 gene expression. These data suggest a functional role for MMP-25 in mouse SP development and are the first to identify a role for a single MMP in mouse SP development.

## Background

Orofacial clefts are some of the most common birth disorders today. Typically, they are disfiguring, can affect respiration, speech, and eating, and require many surgeries to repair. The two main types of orofacial clefts are cleft lip with or without cleft palate (CL/P) and cleft palate (CP) alone. CL/P occurs in approximately 0.2 to 2.3 births per 1000 and CP in 0.1 to 1.1 per 1000 births [1]. CL/P can affect the primary palate (PP), in which the four maxillary incisors are set at the front of the mouth, or both the PP and secondary palate (SP). The SP is posterior to the PP and forms the main barrier between the oral and nasal cavities. Similarly, CP can affect the SP or both the PP and SP.

Development of the SP begins around embryonic day (E) 12.0 in mice and in week six during human gestation. Due to developmental similarities between mice and humans, the mouse is an ideal model animal in which to study SP development. Initially, the SP exists as two shelves that arise from maxillary prominences on either side of the tongue. These shelves will grow downward alongside the tongue then quickly elevate when the tongue depresses, ultimately growing together around E14.0. With their medial edges in contact, the two shelves fuse together and the epithelial cells at the center form the midline epithelial seam (MES). By E15.5-E16.0, this MES has degraded and a solid, confluent SP remains (reviewed in [2,3]). Development of the SP is a carefully coordinated event and requires the actions of many proteins, including transcription factors [4], growth factors and their receptors, and tissue re-modeling enzymes (reviewed in [5]). Any problem with shelf growth, elevation, tongue depression, shelf fusion, or MES degradation can result in a CP.

Among the proteins required for SP development are the matrix metalloproteinases (MMPs). These are a group of proteases with extra-cellular matrix substrates that require coordination of a zinc ion at the centre of the catalytic domain to be active. The MMPs are broadly classed into two categories: the secreted and the membrane-associated. They are synthesized intra-cellularly as pro-enzymes and activated via cleavage of their pro-domain in the Golgi network or extra-cellularly (reviewed in [6]). Treatment of *in vitro *SP cultures with a general chemical inhibitor of the MMPs results in impaired palate shelf fusion and persistence of the medial edge epithelium (MEE) where the shelves make contact [7]. However, the MMP family contains 25 members and which one of the MMPs is playing a functional role in SP development is not clear. One candidate is MMP-25 (Membrane-type (MT) 6-MMP; Leukolysin), as a genetic analysis of human CP patients reveals a potential association between MMP-25 and CL/P [8].

MMP-25 is a membrane-associated MMP first cloned from leukocytes but later found in most tissues examined [9,10]. This protein appears to have roles in both healthy and diseased systems. In healthy systems, MMP-25 can act as both an extra-cellular protease and an activator of some secreted pro-MMPs (reviewed in [11]); however, MMP-25 is also over-expressed in certain cancers and appears to play a role in their progression [12,13].

Despite evidence to support a functional role for MMPs in SP development, no further investigations of individual MMPs and their role in SP formation have been conducted and thus the role of MMP-25 in SP development is currently unknown. To our knowledge, this study represents the first work done on MMP-25 in the mouse SP.

Using quantitative real-time PCR, *in situ *hybridization (ISH), western blot analysis, and immunohistochemistry (IHC), we show MMP-25 mRNA and protein are expressed at all stages of the developing mouse SP with a significant down-regulation at approximately E15.5 as the MES degrades and SP development comes to an end. Using an *in vitro *culture method and MMP-25-specific small, interfering RNA (siRNA), we show *in vitro *SP cultures treated with MMP-25 siRNA exhibit significantly decreased fusion success and increased persistence of the MEE. Lastly, addition of a TGF-β3-neutralizing antibody to *in vitro *SP cultures results in significantly decreased MMP-25 mRNA and protein expression, providing evidence that MMP-25 is a downstream target of TGF-β3 signaling.

## Results and Discussion

### MMP-25 is expressed in the developing SP at all stages

**Figure 1:**
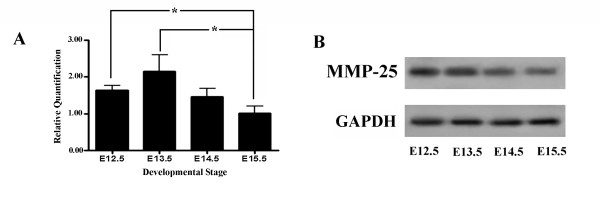
**Matrix metalloproteinase-25 (MMP-25) mRNA and protein are expressed in the developing mouse palate at all stages**. (A) Quantitative real-time PCR indicates a significant down-regulation in MMP-25 mRNA levels at E15.5 compared to E12.5 and E13.5 (*p *< 0.05; n = 4 for all stages). Error bars indicate standard error of the mean. (B) Western blot analysis displays a reduction in MMP-25 protein levels at E15.5 relative to E12.5 and E13.5. GAPDH was used as a loading control.

**Figure 2:**
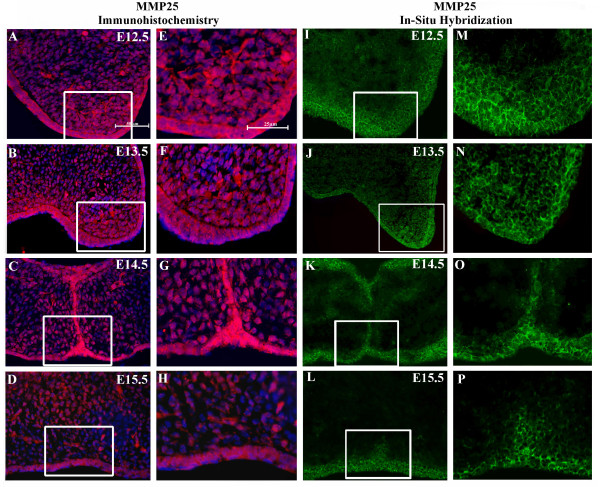
**Localization of matrix metalloproteinase-25 (MMP-25) protein and mRNA expression in the developing mouse palate**. (A-H) Immunofluorescent images of MMP-25 protein expression (red) colocalized with Hoechst nuclear staining (blue). (I-P) *In situ *hybridization of MMP-25 mRNA expression (green). Expression of MMP-25 appears stronger in the epithelium of the palate shelves than in the underlying mesenchyme. (E-H) Enhanced views of highlighted areas from A-D. (M,P) Enhanced views of highlighted areas from I-L. For A-D and I-L, scale bar indicates 50 μm. For E-H and M-P, scale bar indicates 25 μm.

Quantitative real-time PCR and ISH confirm MMP-25 mRNA expression in the embryonic mouse SP at all developmental stages (Figs. 1A, 2I-P). Expression of MMP-25 mRNA increases from E12.5 to E13.5, where its expression is strongest, before decreasing at E14.5 and even further by E15.5. MMP-25 mRNA levels are significantly decreased at E15.5 relative to E12.5 and E13.5 (*p *< 0.05). The increased expression of MMP-25 at E12.5 and E13.5 suggests a role for MMP-25 in promoting palate shelf growth. At this stage in development, the palate shelves are budding from the maxilla and growing downward alongside the tongue. A decline in MMP-25 expression occurs as the shelves are fusing (E14.5) and the MES is degrading (E15.5). Western blot analysis indicates MMP-25 protein expression levels parallel mRNA levels in the embryonic mouse SP (Fig. 1B). MMP-25 appears as a band of approximately 55 kilodaltons (kDa), which corresponds to previously published reports of a 57 kDa molecular weight for MMP-25 [13,14].

### MMP-25 protein and mRNA levels are highest in the epithelium of the palate shelves

IHC and ISH indicate MMP-25 protein and mRNA localize more strongly to the epithelium of the palate shelves than the underlying mesenchyme (Fig. 2). At E12.5 and E13.5, the strongest immunofluorescence is detected in the epithelium of the palate shelves although staining is also visible in the apical mesenchyme (Fig. 2A, E; 2B, F; 2I, M; 2J, N. These two stages represent continued palate shelf growth alongside the tongue before the shelves elevate and make contact along their medial edges. At E14.5, the palate shelves are fusing along their medial edges and immunofluorescence shows strong MMP-25 expression in the epithelium of the palate shelves (Fig. 2C, G; 2K, O) with expression also visible in the underlying mesenchyme. At E15.5, the MES is degrading and little epithelium is left between the two palate shelves (Fig. 2D, H; 2L, P). MMP-25 immunofluorescence at this stage is noticeably weaker than from E12.5 to E14.5, but remains prominently in the epithelium as indicated by the oral epithelium and a degrading remnant of the MES (Fig. 2L, P). This decline in MMP-25 expression at E15.5 coincides with a confirmed decline in MMP-25 mRNA and protein levels (Fig. 1A, B). MMP-25 immunofluorescence in the SP localizes to the plasma membranes of the epithelial and mesenchymal cells, which is expected as MMP-25 is a membrane-associated MMP [15]. The data indicating stronger MMP-25 expression in apical areas of palate shelf growth from E12.5 and E13.5 suggest MMP-25 may play a role in facilitating this growth, possibly by removing extra-cellular matrix barriers to increased cell proliferation and movement and/or by activating pro-MMPs that could do the same [11,16].

### MMP-25 has a functional role during mouse SP formation

**Figure 3:**
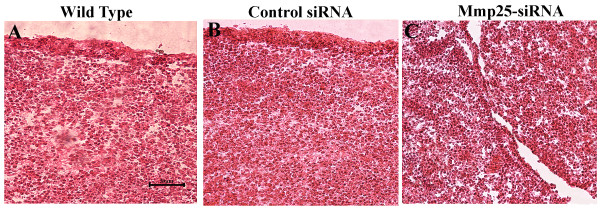
**Representative hematoxylin and eosin staining of embryonic day (E) 13.0 *in vitro *cultured palates after 72 h**. (A) Palate shelves incubated for 72 h with growth medium fused normally. (B) Palate shelves grown with 500 nM scrambled control siRNA fused normally. (C) Palate shelves grown with 500 nM matrix metalloproteinease-25-specific siRNA did not fuse normally and contained considerable medial edge epithelium compared to the control groups.

**Figure 4:**
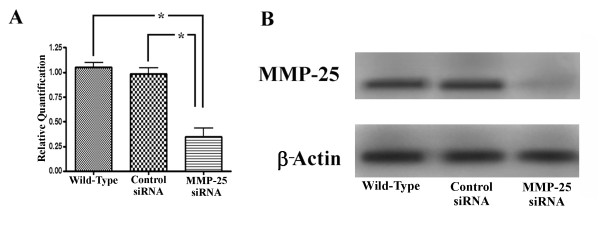
**Confirmation of specific matrix metalloproteinase-25 (MMP-25) knockdown for *in vitro *palatal cultures**. (A) Quantitative real-time PCR shows a significant decrease in MMP-25 mRNA levels from *in vitro *cultures following treatment with 500 nM MMP-25 siRNA (*p *< 0.01). Error bars indicate standard error of the mean. (B) MMP-25 protein expression is reduced in *in vitro *palatal cultures following treatment with MMP-25 siRNA. β-Actin was used as a loading control.

To determine if MMP-25 is playing a functional role during mouse SP formation, MMP-25 expression was knocked down *in vitro *using MMP-25 siRNA and whole SP cultures as previously described [17,18]. Addition of Stealth™ RNAi MMP-25 siRNA (Invitrogen) to a final concentration of 500 nM resulted in decreased palate shelf fusion and persistence of the MEE *in vitro *as evidenced by representative hematoxlyin and eosin-stained palatal sections (Fig. 3). While the wild-type and negative control siRNA palatal cultures fused normally, the palatal cultures treated with MMP-25 siRNA seemed largely unable to progress to the fusion stage despite making contact. Interestingly, the phenotype of our MMP-25 siRNA-treated palatal cultures (Fig. 3C) after 72 h incubation is very similar to the phenotype of other *in vitro *palatal cultures treated with a general chemical inhibitor of the MMPs [7]. To obtain a more objective measure of the MMP-25 siRNA effects, serial sections of preserved and frozen palatal cultures were generated. Sections examined by light microscopy were assigned a score between 1 and 5 based upon fusion success [19] (see Methods). For each palatal culture examined, the scores were combined and averaged to obtain a mean fusion score (MFS) and these MFSs themselves averaged for an entire group (Table 1). The wild-type control palatal cultures (n = 10) had a cumulative MFS of 4.14 while the scrambled duplex siRNA negative control group (n = 10) had a MFS of 4.13. These scores put both groups solidly in the fusion category and indicate minimal non-specific siRNA or transfection reagent effects. The MFS of the palatal culture group treated with 500 nM MMP-25 siRNA (n = 10) was significantly lower at 2.50 (Kruskal-Wallis test; *p *< 0.05). To ensure the drop in MFS could be attributed to MMP-25 mRNA and subsequent protein knockdown, quantitative real-time PCR and western blot analyses were carried out on RNA and protein isolated from the remaining palatal cultures from each group. The MMP-25 siRNA knocked down both MMP-25 mRNA and protein levels efficiently (Fig. 4). Quantitative real-time PCR shows a significant decrease in MMP-25 mRNA levels in the MMP-25 siRNA-treated group relative to the control groups (Fig. 4A; *p *< 0.05), and western blot analysis displays a sharp reduction in MMP-25 protein levels in the treated group when compared to the control groups (Fig. 4B). Taken together, these data suggest a functional role for MMP-25 in mouse SP development.

**Table 1:** Mean fusion scores for palatal cultures.

**Culture**^**a**^	Number	**MFS**^**b**^
Wild-type	10	4.14
Scrambled Control siRNA	10	4.13
MMP-25 siRNA	10	2.50

^a^Palate shelves were removed at E13.0 and cultured for 72 h prior to sectioning and assessment of shelf fusion.

^b^Mean of scores based on the success of fusion as per [19].

### MMP-25 is a downstream transcriptional target of TGF-β3

**Figure 5:**
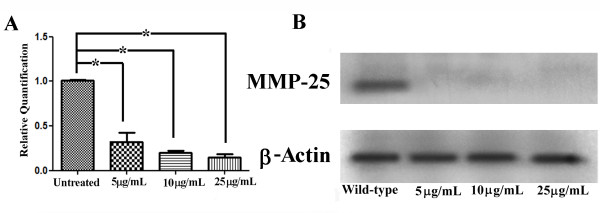
**Matrix metalloproteinase-25 (MMP-25) mRNA and protein expression is decreased in *in vitro *palatal cultures following treatment with a TGF-β3-neutralizing antibody**. (A) Quantitative real-time PCR data indicates a significant reduction in MMP-25 mRNA levels in *in vitro *palatal cultures after exposure to a TGF-β3-neutralizing antibody. (B) MMP-25 protein is decreased upon bio-neutralization of TGF-β3. β-Actin was used as a loading control.

**Figure 6:**
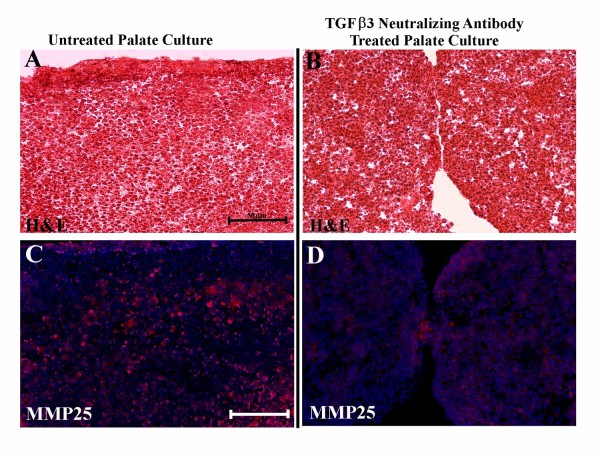
**(A, C) Embryonic day (E) 13.0 *in vitro *cultured control palate after 72 h, (B,D) E13 *in vitro *cultured palate (treated with TGF-β3-neutralizing antibody, 10 μg/mL) after 72 h**. (A,B) Hematoxylin and eosin (H&E) stained sections. (C,D) Immunohistochemistry of MMP-25 (red) colocalized with Hoechst nuclear staining (blue). TGF-β3-neutralizing antibody-treated culture inhibited palatal fusion (B) and showed weaker MMP-25 protein expression (D). Scale bar indicates 50 μm.

**Figure 7:**
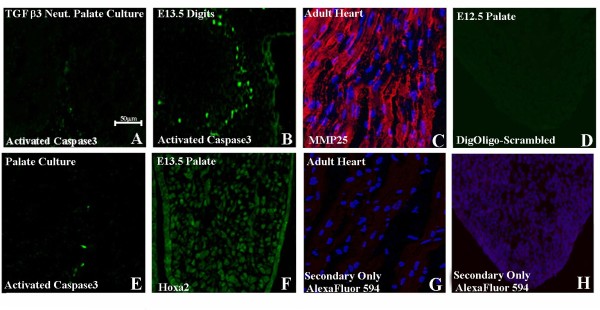
**(A,B,E) Immunohistochemical staining for activated caspase3**. Little or no activated caspase 3 could be identified in control palatal cultures (E) or in TGF-β3-neutralizing antibody-treated palatal culture (A). (B) Section of E13.5 digits exhibiting caspase3 activity as positive control. (C) Immunohistochemistry (IHC) section of adult mouse heart exhibiting MMP-25 expression (red) as positive control colocalized with Hoechst nuclear staining (blue). (G) IHC section of adult mouse heart with only the secondary antibody as negative control. (D) ISH of E12.5 palatal section with scrambled Dig-labeled oligo probe as negative control. (F) IHC of E13.5 palate showing Hoxa2 expression as positive control [4]. (H) IHC of E12.5 palatal culture with only the secondary antibody colocalized with Hoechst nuclear staining (blue) as negative control. Scale bar indicates 50 μm.

**Figure 8:**
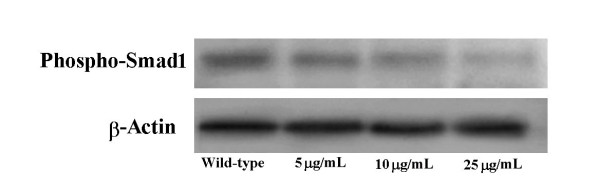
**Western blot analysis against phospho-Smad1 shows a TGF-β3-neutralizing antibody concentration-dependent effect**. Approximately 10 μg of total protein was separated via SDS-PAGE and probed with an anti-phospho-Smad1 antibody (Cell Signaling Technology; 1:500). An anti-β-actin antibody was used to assess loading equivalence (Santa Cruz; 1:1000).

The secreted growth factor TGF-β3 is vital to the successful formation of the mouse SP [20,21]. TGF-β3 exerts biological activity through its receptors, which are tyrosine kinases, and its intra-cellular mediators, the Smad proteins, which translocate to the nucleus affecting gene transcription following phosphorylation (see [22] for a review). An examination of the proximal promoter sequence of MMP-25 obtained from the Eukaryotic Promoter Database http://www.epd.isb-sib.ch/ displayed repeating sequences of 5'-GTCT-3' and 5'-CAGA-3' (data not shown), which are sites of preferential binding by the Smad proteins [23-25]. Due to the existence of Smad binding sites just upstream of the MMP-25 transcription start site and given the important role of TGF-β3 in palate development [20,21], we hypothesized MMP-25 could be a target of TGF-β3. MMP-25 mRNA and protein levels significantly decreased (*p *< 0.05) following 24 h incubation with 5 μg/mL of a TGF-β3-neutralizing antibody (Figs. 5, 6). This decreased expression of MMP-25 is not due to apoptotic cell death as activated caspase3 was not detected in palatal cultures treated with TGF-β3-neutralizing antibody (Fig. 7). MMP-25 mRNA and protein levels between the groups treated with different concentrations of the TGF-β3-neutralizing antibody were not significantly different. To confirm the decrease in MMP-25 mRNA and protein could be attributed to a loss of TGF-β3 activity in the cultures, western blot analysis was carried out on protein isolated from the four groups with a phospho-Smad1 antibody. Levels of phospho-Smad1 were inversely correlated with the concentration of the TGF-β3-neutralizing antibody; as the bio-neutralizing antibody concentration went up, phospho-Smad1 levels went down (Fig. 8).

The strength of the MMP-25 gene expression knockdown by the TGF-β3-neutralizing antibody is supported by several lines of evidence. First, expression of MMP-25 is found in the palate shelf in a manner very similar to the expression pattern of TGF-β3 [26]. Second, TGF-β1 is only detectable after the horizontal palatal shelf stage [26], and expression of TGF-β1 from the TGF-β3 locus cannot completely rescue TGF-β3-induced cleft palate [27]. Spatially, TGF-β2 expression is restricted to the palate mesenchyme and is not apparent until the palate shelves are fusing [26]. Thus, TGF-β2 expression is temporally and spatially distinct from and unlikely to contribute to MMP-25 expression. This evidence also suggests TGF-β3-specific activity in the developing SP that cannot be compensated for by other TGF-β isoforms [27]. A link between the MMPs and TGF-β3 is not without precedent; MMP-13 expression has been demonstrated to be downstream of TGF-β3 [7]. These data suggest MMP-25 is a direct transcriptional target for TGF-β3 in mouse SP development and could be a target for TGF-β3 elsewhere as well.

## Conclusions

MMP-25 mRNA and protein are expressed in the developing mouse SP from stages E12.5 to E15.5, and protein expression largely localizes to the epithelium of the palate shelves. Expression of MMP-25 mRNA and protein is highest at E13.5 and mRNA levels significantly decrease by E15.5. IHC and ISH analysis of MMP-25 protein and mRNA expression, respectively, show MMP-25 is most abundant in the epithelium of the palate shelves although expression is also visible in the underlying mesenchyme. Expression in the mesenchyme appears strongest in apical areas immediately adjacent to the overlying epithelium at E12.5 and E13.5 where it may play a role in facilitating growth. At E14.5, MMP-25 protein and mRNA expression appear highest in medial epithelia (Fig. 2C,G; 2K,O) as palatal shelves come together to fuse. At E15.5, a down-regulation of expression is evident when fusion is nearing completion.

Through the use of palatal cultures, we demonstrate treatment with an MMP-25-specific siRNA decreases palate shelf fusion and results in significant persistence of MEE *in vitro*. This is the first report supporting a functional role for a single MMP in mouse SP development. Our *in vitro *palatal culture work coupled with the IHC, ISH, and real-time PCR data suggest MMP-25 plays a key role in palate shelf growth at E12.5 and E13.5 by removing extra-cellular matrix barriers that would hinder cell proliferation and movement. The substrates of MMP-25 include type IV collagen, fibronectin, and fibrin [28], which are all components of the extra-cellular matrix and barriers to cell proliferation and movement. IHC analysis of MMP-25 protein expression in colorectal tissue sections shows MMP-25 is strongly expressed on the leading edge of tumours and positively correlates to an invasive cancer [13]. Our results indicate MMP-25 protein expression in the developing mouse SP is also higher on the apical, or leading, edge. Most compellingly, double knock-out of MMP-14 (MT1-MMP) and MMP-16 (MT3-MMP) results in a cleft palate in mice due to impaired growth of the palate shelves [29], which implies a role for proteolytic enzymes in mouse SP development even though no palate defects were reported for the single gene knock-out of either MMP-16 [29] or MMP-14 [30]. The protease activity of both MMP-14 and MMP-16 when knocked out on their own may have been compensated for by MMP-25, which is also a proteolytic membrane-associated MMP, just as the collagenolytic activity of MMP-16 was compensated for by MMP-14 in previous experiments [29]. Our results indicate that MMP-25 may be the key proteolytic enzyme among the MMPs during mouse SP formation.

Our treatment of whole palatal cultures with a TGF-β3-neutralizing antibody in the culture medium resulted in a significant decrease in MMP-25 mRNA and protein. Thus, we have established a link between MMP-25 and TGF-β3 in the developing mouse SP that warrants further investigation.

Overall, what is known about MMP-25 in embryonic development remains limited. This is the first report to identify a role for a single MMP in mouse SP development and validates the link between MMP-25 and CL/P identified from human genetic analysis scans of CP patients [8].

## Methods

### Quantitative real-time PCR

All animal procedures were approved by the Animal Research Ethics Board and the Institutional Animal Care and Use Committee of the University of Saskatchewan. Whole palate shelves were dissected from CD1 mouse embryos at the time periods indicated (E12.5-E15.5). Total RNA was isolated using an RNeasy^® ^Mini Kit (Qiagen; Mississauga, ON, Canada) and reverse-transcribed to cDNA using SuperScript™ RNase H-Reverse Transcriptase according to the manufacturer's protocol (Invitrogen; Carlsbad, CA). Gene expression was quantified using the Taqman^® ^primers and labeled probe system (Applied Biosystems; Foster City, CA) and a real-time PCR machine (ABI 7300) from Applied Biosystems. All reactions were performed using the Taqman Universal Master Mix (2X), FAM-labeled Taqman Gene Expression assays for the MMP-25 gene, VIC-labeled Taqman Endogenous Control β-Actin, and 10 ng of cDNA. Thermocycling parameters were as follows: 2 min at 50°C, 10 min at 95°C, 40 cycles of 15 s at 95°C plus 70 s at 60°C. Taqman gene expression assays have all been tested to have efficiencies not significantly different from 1 (Applied Biosystems). However, to ensure that the primer sets were working properly and that multiplexing the gene specific and β-Actin primers did not result in altered amplification efficiencies, the primer sets were examined individually and in complex with β-Actin. Standard curve reactions were run using 1, 10, and 100 ng of palate cDNA and amplification efficiency was calculated using the equation E = 10^(-1/slope standard curve) ^-1 × 100%. In all cases, the efficiency was required to be between 90-110% for both the gene specific and β-Actin primers sets before analysis continued. In most cases, the appropriate efficiencies were possible using 1 μL of the gene specific primer and probe solution and 0.25 μL of the β-Actin primer and probe set per reaction. All comparisons were performed using the relative quantification (RQ) software (Applied Biosystems). For all quantitative real-time PCR, n = 4 with four replicates and error bars indicating the standard error of the mean. Real-time data were normalized to E15.5 (Fig. 1A) or wild-type expression (Figs. 4A, 5A) and expressed as relative quantification values compared to either E15.5 (Fig. 1A) or wild-type/untreated expression (Figs. 4A, 5A).

### Western blot analysis

Total protein was isolated using a modified radioimmunoprecipitation assay (RIPA) buffer protocol. Briefly, palatal tissue was homogenized in RIPA buffer (5 mM ethylenediaminetetraacetic acid, 10 mM TRIS pH 8.0, 0.1% sodium deoxycholate, 0.01% sodium dodecyl sulfate (SDS), 1% Triton X-100, 150 mM sodium chloride, 1 μg/mL aprotinin, 1 μg/mL pepstatin). Homogenized samples were centrifuged at 11,000 rpm at 4°C for 15 min. Supernatant total protein content was quantified using a *DC *Protein Assay Kit (Bio-Rad; Mississauga, ON, Canada) and separated using SDS poly-acrylamide gel electrophoresis with a 10.0% separating gel. Proteins were transferred to a polyvinylidene fluoride membrane and probed for protein expression [4]. Primary antibodies employed were MMP-25 (rabbit polyclonal; Santa-Cruz Biotechnologies, Santa Cruz, CA; 1:1000), β-actin (Santa-Cruz Biotechnologies, Santa Cruz, CA; 1:1000), and GAPDH (rabbit polyclonal; Sigma-Aldrich, Oakville, ON, Canada; 1:15,000).

### Immunohistochemistry

Embryonic mouse heads were preserved in freshly-prepared 4.0% paraformaldehyde (4%PFA) and dehydrated in 30.0% sucrose at 4°C. Frozen coronal sections (8.0 μm) were prepared and placed on microscope cover-slips coated with 0.50% gelatin. IHC with a primary anti-MMP-25 antibody (1:50; Santa Cruz Biotechnology Inc.) was carried out as follows: two washes in 1 × PBS for 30 min each followed by a 20 s exposure to pepsin digest-all solution (Zymed, San Francisco, CA), which was washed off by immersion in the 1 × PBS for an additional 3 min; blocking in solution (3% skim milk, 0.1% Triton X-100 in 1 × PBS) at room temperature for 1.5 h; incubation with 100 μL of the primary antibody, made up in 1 × PBS, for 2 h at room temperature followed by overnight at 4°C; two washes in 1 × PBS for 5 min each; incubation with 100 μL of Alexa Fluor^® ^594 goat anti-rabbit IgG secondary antibody (1:400; Molecular Probes, Eugene, OR), made up in 1× PBS, for 2 h at room temperature in the dark; two washes in 1 × PBS for 5 min each; and mounting onto glass microscope slides using ProLong^® ^Gold antifade reagent (Invitrogen). IHC was evaluated by observing sections on an Olympus BX40 fluorescent microscope (Olympus America Inc., Center Valley, PA) at an emission wavelength of 613 nm. Images were taken using Image-Pro Plus (v. 6.2; MediaCybernetics, Inc., Bethesda, MD). Determination of non-specific staining was assessed by substituting 100 μL of the secondary antibody in lieu of 100 μL of the primary antibody solution (Fig. 7). All preceding and subsequent steps were carried out as described above [4]. Positive control IHC (Fig. 7) was obtained using adult mouse heart sections where MMP-25 expression is high [10].

### *In situ *hybridization

Embryos were fast frozen in isopentane on dry ice and stored at -80°C before use. Methodology for ISH was modified from the GeneDetect^® ^protocol. In brief, palatal tissues were sectioned (8 μm) on poly-L-lysine coated slides and incubated in RNase free 4% PFA (in PBS, pH 7.2) for 10 min followed by rinsing in PBS for 2 × 5 min. This was followed by 10 s treatment with 10 μg/mL of proteinase K and rinsing in PBS for 2 × 5 min. Sections were incubated in 100% ethanol for 5 min and air dried. Sections were prehybridized in hybridization buffer (GeneDetect^®^) at 45°C for 3 h in a humidified chamber. This was followed by incubation in hybridization buffer containing the MMP-25 DIG-labeled (Integrated DNA Technologies) probe (4 ng/μL for total volume of 25 μL per section) at 37°C for 20 h. Sections were washed in the following buffers: 1 × SSC at room temperature (RT) for 2 min and 0.5 × SSC at RT for 2 min × 2. Sections were rinsed in PBS and covered with blocking solution (PBS, 0.1% Triton X-100, 1% sheep serum) for 30 min. This was followed by incubation with anti-Digoxigenin-fluorescein (FITC) secondary antibody (1:50) in blocking solution for 3 h at RT. A final wash for 5 min in 1 × PBS and followed by mounting onto glass microscope cover slides using ProLong^® ^Gold antifade reagent (Invitrogen). Determination of non-specific staining was assessed by substituting 25 μL of DIG-labeled scrambled probe in lieu of 25 μL of the DIG-labeled probe (Fig. 7).

### *In vitro *palatal cultures

Palate cultures were set up as previously described [4,17,18]. Briefly, timed pregnant CD-1 mice were sacrificed at E13.0 and their embryos removed. The palate shelves were aseptically excised from the E13.0 embryos (staged according to [31]) in Hanks' balanced salts solution and placed in pairs in the proper anterior-posterior orientation on Nucleopore filters (Whatman; 8.0 μm) with their medial edges contacting and the oral side down. Shelves were cultured in BGJb medium (Invitrogen) for 72 h at 37.0°C with 5.0% CO_2_. Experimental groups all had n = 5 (number of pregnant female mice from which the embryos were extracted). From these mice, 10 embryos were randomly selected, staged [31], and used for each treatment group for a total of 30 embryos examined (10 each for wild-type, scrambled control siRNA, and MMP-25 siRNA groups).TGF-β3-neutralizing antibody *in vitro *palatal cultures were incubated for 24 h.

### Treatment with siRNA

The transfection of siRNA occurred as previously described [17,18]. siRNA aliquots (500 nM) were produced by diluting a stock siRNA solution with BGJb medium (Invitrogen) containing 0.2% *Trans*IT-TKO^® ^transfection reagent (Mirus Bio, Madison, WI); the total volume was 1.4 mL. The siRNA used was Stealth™ RNAi MMP-25 siRNA (Invitrogen) and Stealth™ RNAi Negative Universal Control siRNA to assess for any non-specific siRNA effects.

### Scoring palate cultures

Following 72 h incubation, palate cultures were preserved with freshly-prepared 4.0% formaldehyde at 4.0°C for 2 h and dehydrated with 30.0% sucrose. Frozen serial sections (8.0 μm) were prepared and evaluated by light microscopy to assess palatal shelf fusion [4]. Every eighth section was examined and up to 15 sections were examined in total. Each section was given a score based on the completeness of fusion and quantity of remaining epithelium, where 1 indicates no contact between the palate shelves and 5 indicates complete fusion as previously described [19]. A total of 10 cultures were evaluated for each of the three groups: wild-type, scrambled control siRNA, and MMP-25 siRNA.

### TGF-β3-neutralizing antibody treatment

A TGF-β3-neutralizing antibody confirmed to be specific to TGF-β3 (R&D Systems, Minneapolis, MN) was added to the BGJb culture medium (Invitrogen) to final concentrations of 0 (wild-type control), 5, 10, and 25 μg/mL. Palatal cultures were incubated at 37°C and 5% CO_2 _for 24 h as previously described [32]. Following incubation, palatal cultures (n = 10) were pooled for RNA and protein isolation prior to quantitative real-time PCR and western blot analysis [4].

## Authors' contributions

GB carried out the initial *in vitro *studies, palatal organ cultures, participated in the design, and drafted the manuscript. AN contributed to the conception, participated in its design and coordination, helped draft the manuscript, and revised it critically for content. Both authors read and approved the final manuscript.
